# Supplementary material for: Quantification of Grapiprant and Its Stability Testing under Changing Environmental Conditions
Source: Biomedicines. 2022 Nov 5;10(11):2821. doi: 10.3390/biomedicines10112821 (PMC9687689; doi:10.3390/biomedicines10112821)
Supplement: Supplementary file 1 [file biomedicines-10-02821-s001.zip › biomedicines-1984581-supplementary.pdf]

biomedicines

MDPI

Supplementary Material

Quantification of grapiprant and its stability testing under changing environmental conditions by TLC-densitometry

Paweł Gumułka [1,2], Monika Tarsa [3], Monika Dąbrowska [1] and Małgorzata Starek [1,*]

[1] Department of Inorganic and Analytical Chemistry, Faculty of Pharmacy, Jagiellonian University Medical College, 9 Medyczna St, 30-688, Kraków, Poland
[2] Doctorial School of Medical and Health Sciences, Jagiellonian University Medical College, 16 Łazarza St, 31-530, Kraków, Poland
[3] Department of Organic Chemistry, Faculty of Pharmacy, Jagiellonian University Medical College, 9 Medyczna St, 30-688, Kraków, Poland

Table S1. Percentage of grapiprant content during degradation at room temperature.

Environment	Incubation time [h]											
	0	24	48	96	192	384	528	720	888	1104	1272	1512
1MHCl	100.00	93.69	83.21	78.74	77.64	75.21	72.95	72.68	68.53	66.26	56.76	38.91
0.5MHCl	100.00	73.44	72.69	68.20	64.94	64.90	64.47	64.42	62.16	61.20	55.04	46.44
0.1MHCl	100.00	88.97	83.79	81.86	81.84	80.50	78.83	76.84	69.79	67.13	58.51	54.70
H_2O	100.00	82.35	79.35	75.56	66.00	62.49	53.76	52.95	52.40	48.04	37.84	37.31
0.1MNaOH	100.00	97.62	91.57	83.43	76.71	75.36	66.18	63.37	54.77	49.20	45.22	42.20
0.5MNaOH	100.00	99.19	97.02	88.99	74.01	68.41	60.26	59.92	54.05	47.49	46.22	42.12
1MNaOH	100.00	92.77	85.64	74.06	66.64	66.24	63.78	57.54	56.94	52.43	49.57	40.94
buffer pH 2	100.00	86.30	83.55	71.67	68.04	63.99	58.67	52.18	50.15	42.56	40.87	31.30
buffer pH 7	100.00	78.90	78.06	79.57	79.49	71.62	66.43	64.50	58.09	55.36	40.10	32.17
buffer pH 8	100.00	86.65	84.30	82.15	79.95	76.82	66.67	64.60	63.03	60.86	50.61	42.13

Table S2. Percentage of grapiprant content during degradation at 70°C.

Environment	Incubation time [h]												
	0	1	2	4	24	72	96	144	192	312	384	504	696
1MHCl	100.00	96.39	92.50	84.09	80.24	68.06	60.94	56.17	55.07	41.09	7.04	3.32	0.00
0.5M HCl	100.00	93.08	84.81	75.69	65.88	58.42	52.52	43.39	38.25	25.35	8.51	3.76	0.00
0.1M HCl	100.00	91.10	87.28	80.95	72.99	44.83	32.96	28.93	13.82	2.50	0.00	0.00	0.00
H_2O	100.00	99.09	96.82	78.67	75.19	75.18	73.25	66.60	48.03	38.97	25.65	11.64	5.31
0.1MNaOH	100.00	99.41	98.09	86.70	83.53	81.56	73.95	65.19	59.53	57.05	50.68	47.26	43.74
0.5MNaOH	100.00	99.80	95.42	88.18	87.79	82.84	76.40	65.21	61.47	60.66	41.83	33.43	28.87
1MNaOH	100.00	97.18	94.44	88.25	84.98	78.77	75.50	73.39	71.08	69.73	41.89	31.59	30.27
buffer pH 2	100.00	97.73	91.39	76.71	73.07	62.33	33.28	21.28	16.13	4.04	0.00	0.00	0.00
buffer pH 7	100.00	91.79	85.50	78.65	74.08	68.63	65.54	65.13	55.25	48.08	24.16	15.95	3.56
buffer pH 8	100.00	99.43	94.35	86.92	83.38	75.62	74.85	70.55	61.92	48.52	32.71	29.84	14.61

Grapiprant Standard Substance:

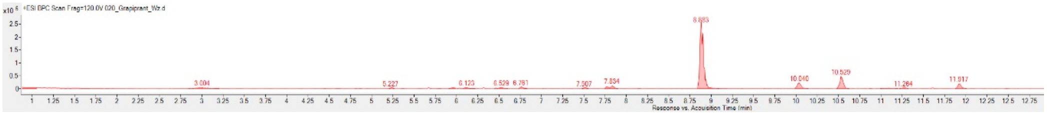

Grapiprant – acidic degradation:

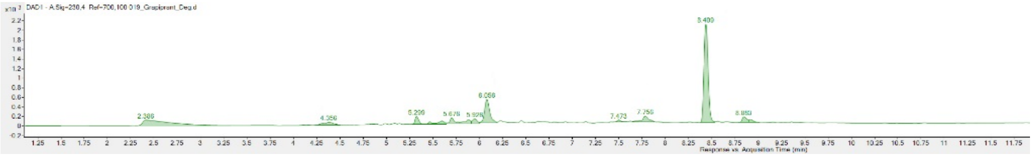

t_r = 8.883 min *m*/*z* 492:

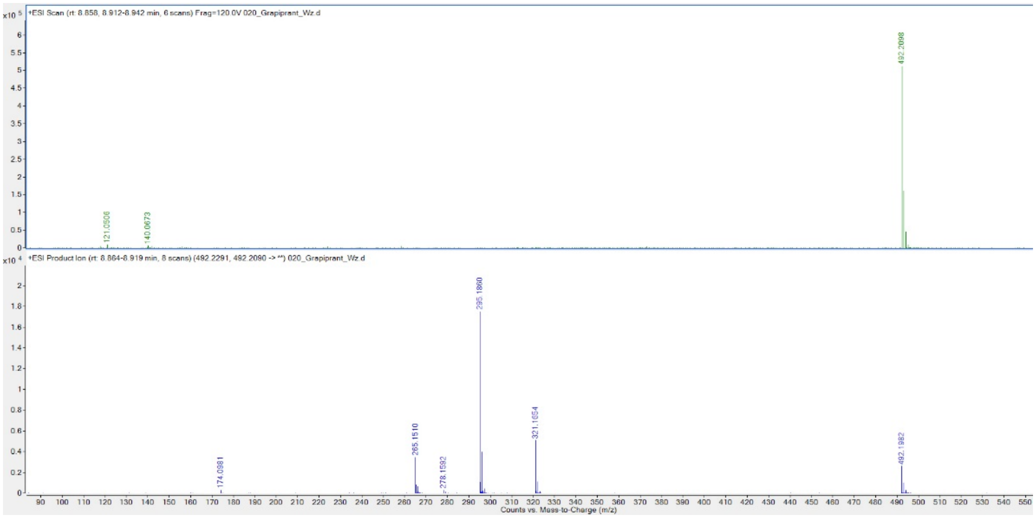

t_r = 4.36 min *m*/*z* 367:

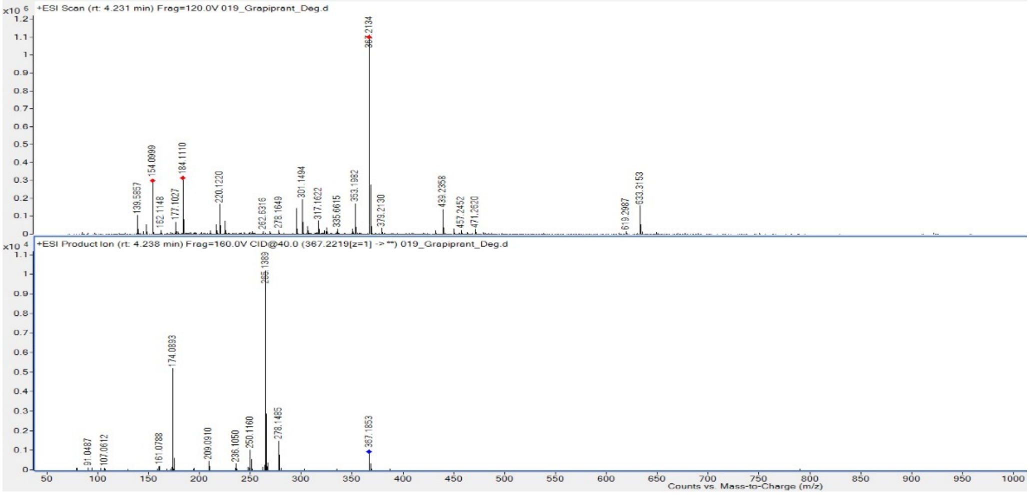

t_r = 6.06 min *m*/*z* 323:

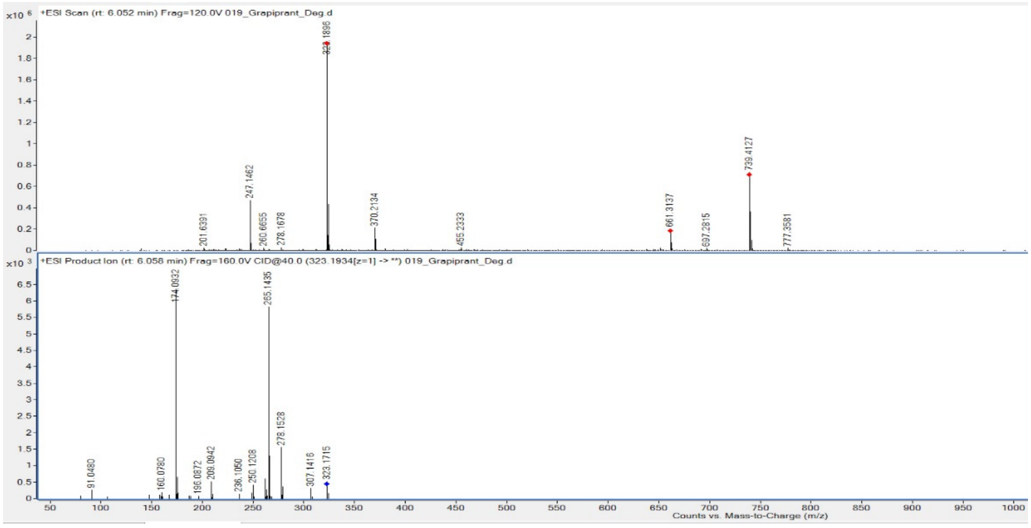

t$_r$ = 2.4 min *m*/*z* 295:

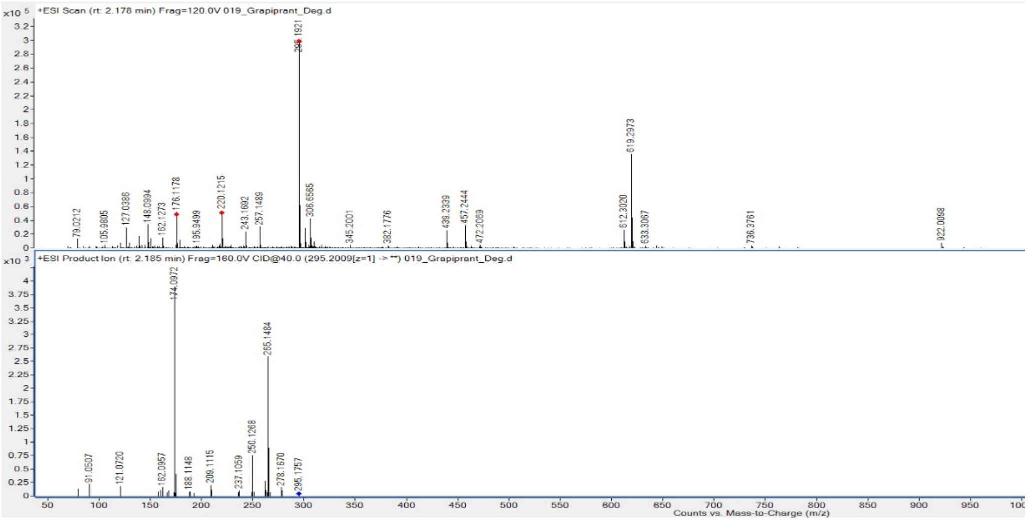

Figure S1. An example of chromatograms and MS spectra registered for samples of grapiprant degradation in acidic solutions.
